# Sacrifício financeiro e capacidade aquisitiva a medicamentos na atenção primária: evidências de um município da Região Metropolitana do Rio de Janeiro, Brasil

**DOI:** 10.1590/0102-311XPT231825

**Published:** 2026-07-27

**Authors:** Alfonso Jesús Gil-López, Letícia Figueira de Castro, Elaine Silva Miranda, Gabriela Silva de Carvalho, Gabriela Bittencourt Gonzalez Mosegui

**Affiliations:** 1 Departamento de Economía y Empresa, Universidad de La Rioja, Logroño, España.; 2 Faculdade de Farmácia, Universidade Federal Fluminense, Niterói, Brasil.; 3 Instituto de Saúde Coletiva, Universidade Federal Fluminense, Niterói, Brasil.

**Keywords:** Atenção Primária à Saúde, Gastos em Saúde, Assistência Farmacêutica, Equidade em Saúde, Acesso a Medicamentos Essenciais, Primary Health Care, Health Expenditures, Pharmaceutical Services, Health Equity, Access to Essential Medicines, Atención Primaria de Salud, Gastos en Salud, Servicios Farmacéuticos, Equidad en Salud, Acceso a los Medicamentos Esenciales

## Abstract

O estudo analisou a prevalência e os fatores associados ao sacrifício financeiro relacionado ao acesso a medicamentos entre usuários da atenção primária em um município da Região Metropolitana do Rio de Janeiro, Brasil. Trata-se de um estudo transversal realizado em 2023 com 290 usuários de unidades de atenção primária de Niterói, distribuídos entre Policlínicas, Programa Médico de Família e Centros de Atenção Psicossocial. O sacrifício financeiro foi definido como a necessidade de adiar compras essenciais, contrair empréstimos ou vender bens para custear despesas com saúde. Aplicaram-se questionários estruturados pautados em métodos validados de avaliação do setor farmacêutico. Realizaram-se análises descritivas e regressão logística binária, com nível de significância de 5%. A prevalência de sacrifício financeiro foi de 35,5% (IC95%: 30,2-41,0), sem diferença significativa entre tipos de serviço (p = 0,416). Internação hospitalar foi o único fator independentemente associado ao desfecho (OR = 2,89; IC95%: 1,05-7,94; p = 0,04). Despesas com medicamentos foram relatadas por 6,2% dos entrevistados, sem associação significativa com o sacrifício financeiro. As estratégias de enfrentamento mais frequentes foram adiamento de contas (14,8%) e endividamento formal ou informal (12,7%), seguidas por redução na compra de alimentos (5,2%). O sacrifício financeiro afeta mais de um terço dos usuários da atenção primária, sendo as internações hospitalares o principal fator associado. Os achados evidenciam lacunas na proteção financeira do Sistema Único de Saúde e reforçam a necessidade de fortalecer políticas de provisão pública de medicamentos e de proteção contra gastos catastróficos.

## Abstract (en)

This study analyzed the prevalence and factors associated with financial sacrifice related to access to medicines in primary care users in a municipality in the Metropolitan Region of Rio de Janeiro, Brazil. This cross-sectional study was carried out in 2023 with 290 users of primary care units in Niterói (distributed among Polyclinics, the Family Medical Program, and Psychosocial Care Centers). Financial sacrifice was defined as the need to postpone essential purchases, take out loans, or sell goods to pay for health expenses. Structured questionnaires were applied based on validated methods to evaluate the pharmaceutical sector. Descriptive analyses and binary logistic regression were performed with a 5% significance level. The prevalence of financial sacrifice totaled 35.5% (95%CI: 30.2-41.0), with no significant difference between types of service (p = 0.416). Hospital admission was the only factor independently associated with the outcome (OR = 2.89; 95%CI: 1.05-7.94; p = 0.04). This study found that 6.2% of interviewees reported expenses with medication, with no significant association with financial sacrifice. The most frequent coping strategies referred to bill postponement (14.8%) and formal or informal indebtedness (12.7%), followed by reduction in food purchases (5.2%). Financial sacrifice affects more than a third of primary care users, with hospital admissions being the main associated factor. These findings highlight financial protection gaps in the Brazilian Unified National Health System and reinforce the need to strengthen policies for the public provision of medication and protection against catastrophic expenditures.

## Abstract (es)

El estudio analizó la prevalencia y los factores asociados al sacrificio económico relacionado con el acceso a los medicamentos entre los usuarios de la atención primaria en un municipio de la Región Metropolitana de Río de Janeiro, Brasil. Se trata de un estudio transversal realizado en el 2023 con 290 usuarios de unidades de atención primaria en Niterói, distribuidos entre Policlínicas, el Programa Médico de Familia y los Centros de Atención Psicosocial. El sacrificio financiero se definió como la necesidad de posponer compras esenciales, solicitar préstamos o vender activos para cubrir los gastos de salud. Se aplicaron cuestionarios estructurados basados en métodos validados de evaluación del sector farmacéutico. Se realizaron análisis descriptivos y regresión logística binaria, con un nivel de significancia del 5%. La prevalencia del sacrificio económico fue del 35,5% (IC95%: 30,2-41,0), sin diferencias significativas entre los tipos de servicio (p = 0,416). La hospitalización fue el único factor asociado de forma independiente con el desenlace (OR = 2,89; IC95%: 1,05-7,94; p = 0,04). El 6,2% de los encuestados declaró haber incurrido en gastos de medicación, sin que existiera una asociación significativa con el sacrificio económico. Las estrategias de afrontamiento más frecuentes fueron el aplazamiento del pago de facturas (14,8%) y el endeudamiento formal o informal (12,7%), seguido de la reducción de la compra de alimentos (5,2%). El sacrificio económico afecta a más de la tercera parte de los usuarios de la atención primaria, y las hospitalizaciones son el principal factor asociado. Los hallazgos ponen de manifiesto las lagunas en la protección económica del Sistema Único de Salud y refuerzan la necesidad de fortalecer las políticas públicas de suministro de medicamentos y de protección contra gastos catastróficos.

## Introdução

O acesso a medicamentos constitui dimensão essencial da cobertura universal em saúde, sendo a disponibilidade e a capacidade aquisitiva elementos estratégicos destacados pela Organização Mundial da Saúde (OMS) ^1^. No marco conceitual de Penchansky & Thomas ^2^, a capacidade aquisitiva (*affordability*) é definida como o ajuste entre o custo dos serviços e a capacidade de pagamento dos usuários, dimensão particularmente relevante em contextos de desigualdade socioeconômica.

No Brasil, apesar de avanços na ampliação do acesso por meio de iniciativas como o Programa Farmácia Popular e políticas de provisão no Sistema Único de Saúde (SUS), persistem desafios. O financiamento público do consumo de medicamentos permanece aquém de referências internacionais, com a média da Organização para Cooperação e Desenvolvimento Econômico (OCDE) em aproximadamente 58% ^3^, já desigualdades regionais e por tipo de serviço são evidentes no território nacional ^4^ ^,^ ^5^. Estimativas nacionais indicam que cerca de 3% dos domicílios brasileiros enfrentam gastos catastróficos com saúde ^6^, definidos pela OMS ^7^ como despesas que ultrapassam 10% ou 25% da capacidade de pagamento familiar, com os medicamentos representando um componente importante dessa despesa, especialmente impactando os estratos mais pobres ^8^.

O conceito de sacrifício financeiro - entendido como a necessidade de adotar estratégias como contrair dívidas, vender bens ou atrasar contas para viabilizar despesas em saúde - permanece pouco explorado em pesquisas nacionais, sobretudo em nível local ^9^ ^,^ ^10^. Esse indicador amplia a compreensão tradicional de gasto catastrófico ao captar estratégias de enfrentamento que, embora não necessariamente atinjam o limiar de 10% ou 25% da renda familiar estabelecido pela OMS ^7^, comprometem a estabilidade financeira dos domicílios e explicitam escolhas forçadas impostas pelo adoecimento.

A literatura internacional documenta que barreiras financeiras ao acesso a medicamentos constituem um problema global, afetando até mesmo os sistemas universais de saúde em países de alta renda ^11^. Nos Estados Unidos, aproximadamente um quinto dos adultos relata não preencher prescrições por razões financeiras ^12^, e na Austrália cresce o contingente que deixa de adquirir medicamentos devido ao aumento do custo de vida ^13^. Tais situações vêm sendo descritas sob o conceito de *financial toxicity*, no qual as despesas com saúde afetam não apenas a adesão terapêutica, mas também a qualidade de vida e a estabilidade financeira das famílias ^14^. No contexto da atenção primária brasileira, embora estudos nacionais tenham caracterizado a disponibilidade de medicamentos essenciais e analisado gastos domiciliares ^15^ ^,^ ^16^, há escassez de investigações que explorem especificamente as estratégias de enfrentamento adotadas pelas famílias diante das despesas com saúde e medicamentos. A compreensão dessa dimensão é fundamental para o aprimoramento das políticas de assistência farmacêutica e para a garantia da equidade no acesso.

Diante desse cenário, o presente estudo teve como objetivo analisar a prevalência e os fatores associados ao sacrifício financeiro relacionado ao acesso a medicamentos entre usuários de serviços ambulatoriais da rede pública municipal em um município da Região Metropolitana do Rio de Janeiro, Brasil, identificando também estratégias de enfrentamento adotadas pelas famílias.

## Métodos

### Desenho do estudo

Estudo transversal integrante do projeto *Modelo de Gestão para o Município de Niterói: A Assistência Farmacêutica que Queremos*, realizado em 2023 ^17^. O trabalho foi conduzido em Niterói, município da Região Metropolitana do Rio de Janeiro, que tem 481.758 habitantes distribuídos em cinco regiões administrativas, apresentando Índice de Desenvolvimento Humano Municipal (IDHM) de 0,837, classificado como muito alto ^18^. A rede pública municipal de saúde é composta por 72 unidades, incluindo 10 Policlínicas, 7 Centros de Atenção Psicossocial (CAPS) e 34 unidades do Programa Médico de Família (PMF) ^19^.

### Participantes do estudo

Participaram 290 usuários maiores de 18 anos, adotou-se amostragem por conveniência. O número de participantes foi definido pela viabilidade operacional e pelo fluxo de usuários atendidos nas unidades de saúde, incluindo todos os indivíduos elegíveis que aceitaram participar da pesquisa durante o período de coleta de dados. Considerando a natureza exploratória da investigação e a inexistência de estimativas prévias de prevalência de sacrifício financeiro na população estudada, utilizou-se o pressuposto conservador de máxima variabilidade (p = 50%) para o cálculo do tamanho amostral. Levando-se em conta o nível de 95% de confiança e o erro absoluto aproximado de 6%, estimou-se um mínimo de cerca de 250 participantes. A amostra efetivamente obtida (n = 290) foi considerada suficiente para descrever a frequência do fenômeno e explorar associações ^20^. A estratégia de recrutamento permitiu abordar usuários recém-expostos ao serviço farmacêutico, aumentando a pertinência das respostas e garantindo a exequibilidade da coleta.

### Instrumentos e procedimentos de coleta de dados

Foram aplicados questionários semiestruturados elaborados com base em método de avaliação da OMS para estudos de acesso a medicamentos ^21^ e na *Pesquisa Nacional sobre Acesso, Utilização e Promoção do Uso Racional de Medicamentos* (PNAUM) ^22^.

A coleta de dados foi realizada por uma equipe de dez pesquisadores, composta por estudantes de graduação e pós-graduação da Universidade Federal Fluminense (UFF), previamente capacitados. O treinamento incluiu palestras sobre assistência farmacêutica, orientações éticas para abordagem dos participantes e instruções sobre os procedimentos e a qualidade da coleta de dados em campo.

As respostas foram registradas na plataforma Research Electronic Data Capture (REDCap), versão 12.4.16, hospedada no site da UFF (https://redcap.uff.br/redcap/index.php), escolhida por sua confiabilidade, segurança na gestão de dados e compatibilidade com dispositivos móveis ^23^. A coleta ocorreu simultaneamente nas cinco regiões administrativas do município ao longo de duas semanas consecutivas.

### Variáveis do estudo

A análise do acesso aos medicamentos seguiu o marco conceitual de Penchansky & Thomas ^2^, considerando cinco dimensões: disponibilidade, capacidade aquisitiva, acessibilidade geográfica, acomodação e aceitabilidade. Para avaliar a dimensão capacidade aquisitiva, foram consideradas as seguintes perguntas:

(1) “No último ano a sua família deixou de comprar algo importante para o dia a dia, precisou fazer algum empréstimo ou vendeu algo para pagar gastos com algum problema de saúde?”. Resposta: sim/não.

(2) “Que tipo de problema ocasionou este gasto?”. Esta questão admitia múltiplas respostas não mutuamente exclusivas, contemplando as seguintes categorias: medicamentos, consulta médica, exame, internação, cirurgia e outros.

(3) “Como foi que a família lidou com esse gasto?”. Trata-se de uma pergunta fechada, de única escolha, cujas respostas incluíram as opções: nenhuma estratégia relatada, adiou pagamento de contas, contraiu empréstimos (bancário ou informal), reduziu compra de alimentos, vendeu bens.

A variável desfecho do estudo foi denominada “sacrifício financeiro”, definida operacionalmente como resposta afirmativa à primeira pergunta, indicando que a família precisou adotar pelo menos uma estratégia de ajuste financeiro para custear despesas relacionadas à saúde nos 12 meses anteriores à pesquisa.

As variáveis independentes incluíram: características sociodemográficas (sexo, idade, escolaridade) e bairro/zona de residência, tipo de serviço de saúde (Policlínica, PMF, CAPS), e tipos específicos de despesa em saúde (medicamentos, consultas médicas, exames, internações, cirurgias e outros). Para fins analíticos, cada categoria de tipo de despesa foi tratada como variável dicotômica independente (sim/não), dada a natureza não mutuamente exclusiva das respostas.

### Análise estatística

Realizaram-se análises descritivas por meio de frequências absolutas e relativas para variáveis categóricas e medianas, com intervalos interquartis para variáveis numéricas, em razão da distribuição assimétrica dos dados. As associações entre variáveis categóricas foram avaliadas mediante teste qui-quadrado de Pearson (χ^2^), após a verificação dos pressupostos de aplicação, considerando-se frequência esperada mínima adequada ≥ 5 em pelo menos 80% das células ^24^. A magnitude das associações foi estimada pelo V de Cramer, adotando-se os seguintes pontos de corte: valores < 0,10 (associação muito fraca); entre 0,10 e 0,29, (fraca); entre 0,30 e 0,49 (moderada) e ≥ 0,50 (forte) ^25^.

A variável referente ao “tipo de despesa em saúde” admitia múltiplas respostas não mutuamente exclusivas. Cada categoria foi analisada separadamente em relação ao sacrifício financeiro, razão pela qual os totais podem exceder n = 290. Células com frequência zero foram mantidas por representarem achados empíricos relevantes. Adicionalmente, foram realizadas análises bivariadas entre as características sociodemográficas e o desfecho. Adotou-se um nível de significância de 5% (p < 0,05). As análises foram realizadas no SPSS 26.0 (https://www.ibm.com/).

### Aspectos éticos

O estudo seguiu as diretrizes da *Resoluções nº 466/2012* ^26^ e *nº 510/2016* ^27^ do Conselho Nacional de Saúde. Os participantes foram informados sobre os objetivos e procedimentos da pesquisa e, após concordarem, assinaram o Termo de Consentimento Livre e Esclarecido. O projeto foi aprovado pelo Comitê de Ética em Pesquisa da Faculdade de Medicina da UFF (CAAE nº 38353120.1.0000.5243, com parecer favorável nº 5.569.440).

## Resultados

Foram analisados 290 usuários de serviços ambulatoriais da rede pública municipal de saúde. Conforme apresentado na Tabela 1, a amostra foi majoritariamente feminina (69%) e composta predominantemente por usuários das Policlínicas (69,3%) seguidas pelo PMF (27,6%) e pelos CAPS (3,1%). Observou-se maior proporção de mulheres entre os usuários do PMF (76,2%), e no CAPS predominou o sexo masculino (66,7%), considerando-se o reduzido número de participantes deste serviço. A idade média dos participantes foi de 50,5 anos, com mediana de 52 anos. As demais características sociodemográficas da amostra estão descritas na Tabela 1.

**Tabela 1:** Características sociodemográficas dos participantes segundo tipo de serviço. Niterói, Rio de Janeiro, Brasil.

Tipo de serviço	Sexo (%)		Idade (anos)				Total	
	Feminino	Masculino	Média	DP	Mediana	Intervalo interquartil	n	%
CAPS	33,3	66,7	44,0	11,3	45	38-50	9	3,1
PMF	76,2	23,8	47,1	15,9	49	34-59	80	27,6
Policlínica	67,7	32,3	52,2	15,5	53	40-64	201	69,3
Total	69,0	31,0	50,5	15,6	52	38-62	290	100,0

CAPS: Centro de Atenção Psicossocial; DP: desvio padrão; PMF: Programa Médico de Família.

Fonte: elaboração própria.

A Tabela 2 apresenta a distribuição do desfecho sacrifício financeiro segundo o tipo de serviço de saúde. Entre os participantes (n = 290), 61,4% não relataram sacrifício financeiro, 35,5% relataram sacrifício financeiro e 3,1% informaram não saber responder. As proporções de sacrifício financeiro foram semelhantes entre os usuários do PMF (36,2%) e das Policlínicas (36,3%). No CAPS, observou-se menor proporção (11,1%), considerando-se o reduzido número de participantes. Não houve associação estatisticamente significativa entre o tipo de serviço e o desfecho (p = 0,416), com tamanho de efeito muito baixo (V de Cramer = 0,082).

**Tabela 2:** Prevalência de sacrifício financeiro segundo tipo de serviço. Niterói, Rio de Janeiro, Brasil.

Tipo de serviço	N total	Sacrifício financeiro				χ^2^		Valor de p		V de Cramer	IC95%
		Sem		Com		NSI					
		n	%	n	%	n	%				
CAPS	9	7	77,8	1	11,1	1	11,1	3,936	0,416	0,082	0,046-0,233
PMF	80	49	61,3	29	36,2	2	2,5				
Policlínica	201	122	60,7	73	36,3	6	3,0				
Total	290	178	61,4	103	35,5	9	3,1				

CAPS: Centro de Atenção Psicossocial; IC95%: intervalo de 95% de confiança; NSI: não sabe informar; PMF: Programa Médico de Família.

Fonte: elaboração própria.

A Tabela 3 apresenta a associação entre as características sociodemográficas e o desfecho sacrifício financeiro. Não foi observada evidência de associação estatisticamente significativa entre o desfecho e as variáveis sexo (p = 0,626; V de Cramer = 0,057), faixa etária (p = 0,198; V = 0,102), escolaridade (p = 0,308; V = 0,114) e bairro/zona de residência (p = 0,176; V = 0,141), com tamanhos de efeito baixos em todas as comparações.

**Tabela 3:** Associação entre características sociodemográficas e sacrifício financeiro. Niterói, Rio de Janeiro, Brasil.

Variável	N total	Sacrifício financeiro				χ^2^		Valor de p		V de Cramer	IC95%
		Sem		Com		NSI					
		n	%	n	%	n	%				
Sexo								0,937	0,626	0,057	0,017-0,197
Feminino	200	125	62,5	70	35,0	5	2,5				
Masculino	90	53	58,9	33	36,7	4	4,4				
Faixa etária (anos)								6,012	0,198	0,102	0,059-0,201
18-39	78	41	52,6	34	43,6	3	3,8				
40-59	123	84	68,3	37	30,1	2	1,6				
60-84	89	53	59,6	32	36,0	4	4,5				
Escolaridade								7,145	0,308	0,114	0,000-0,160
Até Ensino Médio	117	74	63,2	36	30,8	7	6,0				
Ensino Médio	132	79	59,8	52	39,4	1	0,8				
Acima do Ensino Médio	39	24	61,5	14	35,9	1	2,6				
NSI	2	1	50,0	1	50,0	0	0,0				
Zona/Bairro								11,481	0,176	0,141	0,000-0,184
Praias da Baía	100	61	61,0	37	37,0	2	2,0				
Norte	108	72	66,7	31	28,7	5	4,6				
Oceânica	19	13	68,4	6	31,6	0	0,0				
Pendotiba	59	28	47,5	29	49,2	2	3,4				
Leste	4	4	100,0	0	0,0	0	0,0				
Total	290	178	61,4	103	35,5	9	3,1				

CAPS: Centro de Atenção Psicossocial; IC95%: intervalo de 95% de confiança; NSI: não sabe informar; PMF: Programa Médico de Família.

Fonte: elaboração própria.

A Tabela 4 apresenta a associação entre os tipos de despesa em saúde e o desfecho sacrifício financeiro. Observou-se associação estatisticamente significativa entre o desfecho e os gastos relacionados à consulta médica (p = 0,019; V de Cramer = 0,120) e à internação clínica (p = 0,018; V = 0,131), ambas com tamanho de efeito fraco.

**Tabela 4:** Tipos de despesa em saúde e associação com sacrifício financeiro. Niterói, Rio de Janeiro, Brasil.

Tipo de despesa	Total		Sacrifício financeiro			χ^2^ *	Valor de p		V de Cramer **	IC95%
			Sem		Com					
	n	%	n	%	n	%				
Medicamentos	18	6,2	15	83,3	3	16,7	1,08	0,299	0,02	0,00-0,18
Consulta médica	15	5,2	15	100,0	0	0,0	5,46	0,019	0,12	0,02-0,25
Exame laboratorial	107	36,9	70	65,4	37	34,6	1,14	0,286	0,02	0,00-0,18
Internação clínica	74	25,5	43	58,1	31	41,9	5,56	0,018	0.13	0,02-0,25
Cirurgia	52	17,9	33	63,5	19	36,5	0,82	0,365	0,05	0,00-0,17
Outros	52	17,9	35	67,3	17	32,5	0,06	0,810	0,01	0,00-0,12

IC95%: intervalo de 95% de confiança.

Fonte: elaboração própria.

Nota: (i) os totais não somam n = 290 porque as variáveis admitiam múltiplas respostas; (ii) frequência n = 0 em “Consulta médica” indica ausência de casos de sacrifício financeiro atribuídos a esta categoria.

* Teste qui-quadrado de Pearson aplicado separadamente para cada categoria de despesa; p < 0,05;

** V de Cramer: valores < 0,10 = associação muito fraca; 0,10-0,29 = fraca; 0,30-0,49 = moderada; ≥ 0,50 = forte.

Para a categoria consulta médica, não foram observados casos de sacrifício financeiro, o que contribui para a significância estatística observada. Entre os participantes que relataram internação clínica, a proporção de sacrifício financeiro foi maior (41,9%) em comparação às demais categorias de despesa, que não apresentaram associação estatisticamente significativa com o desfecho (p > 0,05).

As estratégias de enfrentamento relatadas pelos participantes diante das despesas em saúde estão apresentadas na Tabela 5. A maioria dos respondentes (66,2%; IC95%: 60,6-71,4) não relatou ações específicas, achado que pode refletir tanto estabilidade financeira quanto possível subdeclaração ou naturalização das dificuldades econômicas. Entre aqueles que relataram estratégias, destacaram-se o adiamento de pagamento de contas (14,8%; IC95%: 11,0-19,6) e a contração de empréstimos bancários ou informais (12,7%; IC95%: 9,3-17,2), seguidos pela redução na compra de alimentos (5,2%; IC95%: 3,1-8,5) e pela venda de bens (1,0%; IC95%: 0,3-3,0).

**Tabela 5:** Estratégias de enfrentamento das despesas relacionadas à saúde. Niterói, Rio de Janeiro, Brasil.

Estratégia adotada	n	%	IC95%
Nenhuma estratégia relatada	192	66,2	60,6-71,4
Adiou pagamento de contas	43	14,8	11,0-19,6
Contraiu empréstimo (bancário ou informal)	37	12,7	9,3-17,2
Reduziu compra de alimentos	15	5,2	3,1-8,5
Vendeu bens	3	1,0	0,3-3,0
Total	290	100,0	

IC95%: intervalo de 95% de confiança.

Fonte: elaboração própria.

Nota: as categorias possuem resposta única.

## Discussão

Este estudo identificou a prevalência de 35,5% de sacrifício financeiro relacionado a despesas com saúde entre os usuários de serviços ambulatoriais da rede pública municipal de Niterói, município de elevado IDHM (0,837) da Região Metropolitana do Rio de Janeiro. Esse achado é particularmente relevante considerando-se que aproximadamente um terço dos usuários de um sistema universal de saúde relatou algum grau de comprometimento da estabilidade econômica familiar associado a gastos com saúde, evidenciando lacunas persistentes na proteção financeira oferecida pelo SUS.

A magnitude observada é compatível com estimativas nacionais que indicam que entre 3% e 5% dos domicílios brasileiros enfrentam gastos catastróficos em saúde, definidos como despesas que ultrapassam 10% ou 25% da capacidade de pagamento familiar ^6^ ^,^ ^8^. O conceito de sacrifício financeiro, entretanto, amplia essa perspectiva ao captar situações nas quais, mesmo sem atingir o limiar catastrófico tradicional estabelecido pela OMS ^11^, as famílias precisam adotar estratégias de enfrentamento que comprometem a qualidade de vida e a segurança econômica.

Estudos internacionais demonstram que aproximadamente 800 milhões de pessoas globalmente incorrem em gastos catastróficos em saúde anualmente, com maior impacto sobre as populações de países de baixa e média renda ^11^. No contexto brasileiro, análises da *Pesquisa Nacional de Saúde* (PNS) de 2019 revelaram que as desigualdades no acesso a medicamentos permanecem expressivas, com maior vulnerabilidade entre populações de menor renda e escolaridade ^5^. Esses achados reforçam que, apesar dos avanços na universalização do acesso à saúde, barreiras econômicas continuam a representar um desafio relevante para a proteção financeira das famílias, ainda que este estudo não tenha identificado diferenças estatisticamente significativas segundo características sociodemográficas.

A internação hospitalar apresentou associação estatisticamente significativa com sacrifício financeiro nas análises bivariadas (p = 0,018; V de Cramer = 0,13), evidências da literatura de que episódios de hospitalização estão frequentemente associados a gastos elevados em saúde e podem comprometer a capacidade de pagamento das famílias. A análise da *Pesquisa de Orçamentos Familiares* (POF) de 2017-2018 demonstrou que internações podem representar até 40% dos gastos totais em saúde das famílias mais pobres ^15^. Esses achados reforçam que, mesmo em contextos de cobertura universal, os custos diretos e indiretos associados a eventos agudos e imprevistos permanecem como importante fonte de risco financeiro, particularmente devido a despesas com transporte, alimentação de acompanhantes, perda de renda por afastamento do trabalho e eventual necessidade de aquisição de medicamentos, insumos e equipamentos não disponibilizados pelo sistema público ^6^.

A ausência de associação estatisticamente significativa entre despesas com medicamentos e sacrifício financeiro nas análises bivariadas merece contextualização cuidadosa. Embora apenas 6,2% dos participantes tenham relatado despesas especificamente com medicamentos, estudos nacionais recentes indicam que os medicamentos representam aproximadamente de 20% a 30% dos gastos privados em saúde das famílias brasileiras, com peso desproporcional sobre os quintis de menor renda ^16^ ^,^ ^28^. Contudo, esses estudos referem-se à população geral, já este estudo focalizou usuários do SUS, população com maior acesso a medicamentos fornecidos gratuitamente pelo sistema público. Nesse sentido, a baixa frequência observada pode refletir particularidades do município investigado, como a existência de uma rede de assistência farmacêutica relativamente estruturada em comparação à média nacional, conforme evidenciado em estudos prévios sobre disponibilidade de medicamentos essenciais na atenção primária ^29^.

Adicionalmente, a ausência de associação pode estar relacionada a limitações inerentes ao desenho transversal, que não permite capturar a cronicidade e a recorrência dos gastos medicamentosos ao longo do tempo. Diferentemente de internações hospitalares, que constituem eventos agudos e facilmente recordados, as despesas com medicamentos para doenças crônicas ocorrem de forma contínua e podem ser subestimadas ou naturalizadas pelos entrevistados como parte do orçamento doméstico regular. Estudos longitudinais demonstram que o ônus financeiro cumulativo dos medicamentos de uso contínuo pode ser substancial, especialmente para portadores de múltiplas condições crônicas ^9^ ^,^ ^10^.

Outro aspecto relevante relaciona-se à disponibilidade de medicamentos na rede pública e às políticas de subsídio implementadas no período do estudo. O Programa Farmácia Popular, vigente à época da coleta de dados, oferecia medicamentos para hipertensão, diabetes e asma de forma gratuita, além de contraceptivos e medicamentos para dislipidemia com copagamento ^4^. Essa estratégia pode contribuir para mitigar o impacto financeiro direto associado à aquisição dos medicamentos para condições crônicas prevalentes. Embora o presente estudo não tenha como objetivo avaliar o impacto desse Programa, a literatura aponta que a ampliação do acesso a medicamentos essenciais por meio de subsídios públicos pode reduzir o gasto direto das famílias e redistribuir o ônus financeiro para outras categorias de despesa em saúde ^3^. Outrossim, estudo anterior documentou redução da proteção financeira após modificações no Programa, com descontinuação de itens e fechamento de unidades da rede própria ^3^.

As estratégias de enfrentamento relatadas - adiamento de contas (14,8%) e contração de empréstimos (12,7%) − evidenciam a necessidade de reorganização do orçamento familiar diante de despesas em saúde. Essas decisões refletem vulnerabilidade estrutural e amplificam desigualdades, comprometendo não apenas a segurança financeira, mas também a continuidade do cuidado e a adesão terapêutica. A literatura internacional documenta que pacientes que enfrentam barreiras financeiras apresentam até 50% mais riscos de não aderir ao tratamento medicamentoso prescrito, com consequências negativas para desfechos de saúde e aumento de custos evitáveis no sistema ^12^.

Entre as estratégias de enfrentamento apontadas, a redução na compra de alimentos, relatada por 5,2% dos participantes, representa estratégia particularmente preocupante, pois evidencia *trade-offs* entre despesas com saúde e segurança alimentar, com potenciais impactos sobre a nutrição e o bem-estar familiar, especialmente em contextos de maior vulnerabilidade socioeconômica. A POF 2017-2018, que introduziu a *Escala Brasileira de Insegurança Alimentar* (EBIA), demonstra correlação inversa entre gastos com saúde e adequação alimentar, especialmente em domicílios de baixa renda ^30^.

Além das estratégias individuais de enfrentamento, a análise segundo bairro/zona de residência não identificou associação estatisticamente significativa com a ocorrência de sacrifício financeiro. Esse resultado deve ser interpretado com cautela, uma vez que a categorização por áreas geográficas agregadas pode não captar adequadamente desigualdades socioeconômicas intraurbanas, especialmente em municípios com elevada heterogeneidade social. Estudos prévios indicam que análises baseadas em unidades territoriais amplas tendem a atenuar gradientes de vulnerabilidade observáveis em escalas mais finas, o que pode limitar a detecção de associações territoriais em estudos transversais. Ainda assim, o achado sugere que o sacrifício financeiro relacionado a despesas em saúde pode afetar usuários residentes em diferentes áreas do município ^31^ ^,^ ^32^.

É relevante mencionar que dois terços dos participantes (66,2%) não relataram estratégias específicas de enfrentamento. Esse resultado ambíguo pode indicar tanto estabilidade financeira quanto subdeclaração por viés de desejabilidade social ou naturalização das dificuldades econômicas como parte inerente do processo de adoecimento, fenômeno documentado em estudos sobre acesso a medicamentos no Brasil ^33^ ^,^ ^34^.

A ausência de diferenças significativas na prevalência de sacrifício financeiro entre os tipos de serviço de saúde indica que este fenômeno esteve presente entre usuários atendidos em diferentes modelos organizacionais. Esse resultado deve ser interpretado com cautela, especialmente diante do número reduzido de participantes em alguns serviços. O achado contrasta com evidências que apontam variações na disponibilidade de medicamentos segundo tipo de serviço ^29^, sugerindo que outros fatores, como perfil socioeconômico dos usuários e padrões de morbidade, podem influenciar a ocorrência de sacrifício financeiro de forma independente das características organizacionais das unidades.

Este estudo apresenta limitações que devem ser consideradas na interpretação dos achados. O delineamento transversal permite identificar associações, mas não estabelecer relações causais ^10^. A amostragem por conveniência e a realização do estudo em um único município limitam a generalização dos resultados, especialmente diante da heterogeneidade regional da oferta de serviços e da assistência farmacêutica no país. O número reduzido de participantes em alguns serviços, como os CAPs, também impõe cautela na interpretação das comparações entre os tipos de serviço ^29^.

As informações autorrelatadas estão sujeitas a viés de memória e de desejabilidade social, particularmente no que se refere às estratégias de enfrentamento dos gastos em saúde ^21^ ^,^ ^22^. Além disso, o desfecho de sacrifício financeiro foi mensurado de forma binária, sem quantificação monetária das despesas, o que dificulta comparações diretas com estudos baseados em medidas de gasto catastrófico. A ausência de informações detalhadas sobre renda domiciliar, cobertura por planos privados e número de dependentes impossibilitou análises mais aprofundadas de subgrupos e o controle de potenciais fatores de confusão.

Apesar dessas limitações, o estudo contribui ao revelar a dimensão subjetiva e econômica do acesso a medicamentos entre usuários do SUS, ao identificar a ocorrência de sacrifício financeiro mesmo em um contexto urbano de elevado IDHM. Ao empregar o conceito de “sacrifício financeiro” como indicador sensível de capacidade aquisitiva, amplia-se a compreensão das desigualdades de acesso no SUS, oferecendo subsídios relevantes para o monitoramento da equidade e da efetividade das políticas de assistência farmacêutica.

Os resultados reforçam a necessidade de políticas integradas que articulem assistência farmacêutica, proteção social e fortalecimento de serviços ambulatoriais da rede pública municipal, com vistas a mitigar o impacto financeiro do adoecimento sobre as famílias. Estratégias voltadas à garantia do abastecimento regular de medicamentos essenciais e ao enfrentamento dos custos associados a eventos de saúde agudos permanecem centrais para a consolidação do direito à saúde e para a redução das desigualdades no acesso aos serviços.

## Conclusões

Este estudo utiliza o conceito de sacrifício financeiro, evidenciando que mais de um terço (35,5%) dos usuários dos serviços ambulatoriais da rede pública em um município socioeconomicamente desenvolvido enfrentou comprometimento da estabilidade econômica por despesas com saúde. Esse indicador, que transcende o gasto catastrófico tradicional, captura dimensões subjetivas e estratégias de enfrentamento que, embora não necessariamente atinjam limiares porcentuais definidos por organismos internacionais, impactam significativamente a qualidade de vida e a segurança financeira dos domicílios.

A associação observada entre internação hospitalar e sacrifício financeiro destaca a relevância de eventos agudos e imprevistos como importantes fontes de risco econômico para usuários do SUS, mesmo em contextos de cobertura universal. Esse achado sugere que custos diretos e indiretos relacionados à hospitalização permanecem como desafio para a proteção financeira das famílias.

As estratégias de enfrentamento relatadas - como adiar contas, endividar-se e reduzir a compra de alimentos - evidenciam restrições orçamentárias associadas ao adoecimento e seus potenciais impactos sobre o bem-estar e a continuidade do cuidado. A ausência de associação estatisticamente significativa entre despesas com medicamentos e sacrifício financeiro não deve ser interpretada como ausência de ônus econômico, devendo ser compreendida à luz do desenho transversal do estudo, de especificidades contextuais e da organização local da assistência farmacêutica.

Os resultados apontam para a urgência de políticas de proteção financeira para eventos de alto custo, como internações, e de ampliação da provisão gratuita de medicamentos nos serviços ambulatoriais da rede pública municipal. A consolidação de programas de abastecimento, a manutenção e a expansão de políticas de subsídio como o Programa Farmácia Popular, e o desenvolvimento de estratégias de mitigação de custos diretos e indiretos associados a hospitalizações são essenciais para que o direito constitucional à saúde seja traduzido em proteção real contra o empobrecimento por despesas com saúde. A utilização do sacrifício financeiro como indicador sensível pode contribuir para o monitoramento da equidade e para o aprimoramento de políticas públicas voltadas à mitigação do impacto econômico do adoecimento, especialmente no que se refere a eventos de maior custo e à garantia do abastecimento regular de medicamentos essenciais.

Estudos futuros, preferencialmente longitudinais e multicêntricos, poderão aprofundar a compreensão dos determinantes do sacrifício financeiro e subsidiar estratégias mais responsivas de proteção econômica em saúde, contribuindo para a consolidação dos princípios da universalidade, integralidade e equidade no SUS.

## Data Availability

Os dados de pesquisa estão disponíveis mediante solicitação à autora de correspondência.
